# Revision of trapeziometacarpal arthroplasty: risk factors, procedures and outcomes

**DOI:** 10.1080/17453674.2019.1599253

**Published:** 2019-04-01

**Authors:** Simo Mattila, Eero Waris

**Affiliations:** Department of Hand Surgery, Helsinki University Central Hospital, Finland

## Abstract

Background and purpose — Revision surgery after trapeziometacarpal arthroplasty is sometimes required. Varying revision rates and outcomes have been reported in rather small patient series. Data on risk factors for revision surgery, on the final outcome of revision, and possible factors affecting the outcome of revision are also limited. We evaluated these factors in 50 patients.

Patients and methods — From 1,142 trapeziometacarpal arthroplasties performed during a 10-year period, 50 patients with 65 revision surgeries were retrospectively identified and invited to participate in a follow-up study involving subjective, objective, and radiologic evaluation. The revision rate, risk factors for revision, and factors affecting the outcome of revision were analyzed.

Results — The revision rate was 5%. Scaphometacarpal impingement was the most common reason for revision surgery. Patient age ≤ 55 years was a risk factor with a revision rate of 9% in this age group, whereas an operation on both thumbs during the follow-up period was a negative risk factor for revision surgery. There was no difference in revision risk between ligament reconstruction and tendon interposition with or without a bone tunnel. 9 patients had multiple revision procedures and their final outcome did not differ significantly from patients revised only once. Most of the patients felt subjectively that they had benefited from revision surgery and the subjective outcome measures (QuickDash and pain VAS) and the Conolly score were in the same range as previously described for revision trapeziometacarpal arthroplasty.

Interpretation — Age ≤ 55 years is a risk factor for revision surgery. The type of primary surgery does not affect the risk of revision surgery and multiple revision procedures do not result in worse outcomes than cases revised only once. Mechanical pain caused by contact between the metacarpal and scaphoid is the most common indication for revision surgery. In general, patients seem to benefit from revision surgery for trapeziometacarpal osteoarthritis.

Osteoarthritis (OA) of the trapeziometacarpal (TMC) joint is a common degenerative disorder frequently treated surgically. Partial or complete trapeziectomy alone or combined with ligament reconstruction and/or tendon interposition (LRTI) are the most commonly used surgical methods, but also various implant procedures have been described (Muermans and Coenen 1998, Richard et al. 2014). In general, surgery for TMC OA is well tolerated with few complications and high patient satisfaction (Wajon et al. 2015). However, some patients may present with persistent or recurrent symptoms such as pain and hand dysfunction. In these cases, revision surgery is considered (Cooney et al. 2006, Megerle et al. 2011). Studies have reported various revision techniques including autologous or alloplastic interposition, ligament reconstruction, suspension, conversion to fusion, and re-excision arthroplasty with varying results (Conolly and Rath 1993, Cooney et al. 2006, Megerle et al. 2011, Papatheodorou et al. 2017, Renfree and Dell 2002, Wilkens et al. 2017). Risk factors for revision surgery have been analyzed in only 2 previous studies (Cooney et al. 2006, Wilkens et al. 2017). So far, no factors have been identified to affect the outcome of patients after revision surgery.

We evaluated the incidence of failed TMC arthroplasty resulting in revision procedures, searched for risk factors for revision surgery, searched for factors affecting the results of revision surgery, and analyzed the final subjective and objective outcomes of revised patients. To our knowledge, this is the largest patient cohort to date on revision TMC arthroplasties.

## Patients and methods

We performed a retrospective chart review to search for all arthroplasty procedures on the thumb trapeziometacarpal (TMC) joint performed during a 10-year period from January 2003 to December 2013 at the single hand surgical unit of Helsinki University Hospital, Finland. The indications for the primary procedures were pain related to primary (Eaton–Glickel stages 1–4) (n = 1,133) or posttraumatic (n = 9) osteoarthritis of the carpometacarpal (CMC-1) joint. Patients with rheumatoid arthritis and patients having had CMC-1 or scaphotrapeziotrapezoidal fusion were excluded from the study. Arthroplasties performed with implants were also excluded due to the small number of patients (n = 32). A consecutive series of 930 patients with 1,142 primary TMC arthroplasties was identified (Table 1).

**Table 1.** Patient demographics

Patients and procedures	
No. of patients	930
Sex (male/female)	121/809
Mean age	61 (34–92)
Primary procedures	1,142
Bilateral procedures	212
Revisions	
No. of patients	50 (45 female)
Mean age	57 (43–80)
Revision procedures	65
Primary revision procedures	52
Cases with multiple revisions	9
Follow-up	
Patients available	38
Mean follow-up, months	43 (8–132)

The following data regarding the primary procedures were collected: age, sex, operated side, arthroplasty performed on both sides, surgeon experience (resident/senior hand surgeon), postoperative immobilization time, type of surgery, simultaneous surgery on the metacarpophalangeal (MCP) joint, other simultaneous surgeries, and postoperative complications.

The patients were divided into 3 groups based on the primary procedure: (1) trapeziectomy and LRTI with the abductor pollicis longus (APL) tendon either through a bone tunnel in the base of the metacarpal (LRTI + bone tunnel group) (Kaarela and Raatikainen 1999) or (2) LRTI with APL without a bone tunnel (a slip of the APL tendon weaved between the remaining APL tendon and flexor carpi radialis (FCR) tendon) (LRTI group) as described by Ceruso et al. (1991). The 3rd group consisted of simple trapeziectomy, partial trapeziectomy with interposition of palmaris longus tendon, LRTI with FCR (Weilby 1988), LRTI with extensor pollicis brevis, or total trapeziectomy and tendon interposition with the palmaris longus tendon without ligament reconstruction (Dell et al. 1978).

From the medical records, 50 patients who had undergone revision surgery were identified and invited for a follow-up visit. The indication for revision surgery, the number of revision procedures performed for each hand, and the techniques of the revision procedures were determined from the medical records. Pre-revision radiographs were available for 42 hands. They were reviewed for the minimum distance between the base of the first metacarpal and scaphoid (scaphometacarpal space) seen in the posteroanterior view, residual bone fragments in the operative area, and MCP-joint hyperextension.

38 of the 50 patients attended the follow-up visit. The mean time from revision to follow-up was 43 months (8–132). Subjective assessment was performed with the Quick Disabilities of the Hand Shoulder and Arm score (QuickDASH), patient evaluation measure (PEM), and the visual analog score for pain (pain VAS). Objective assessment included grip strength with the Jamar Hand Dynamometer (Saehan Corporation, Seoul, South Korea), key and tip pinch strength with the pinch gauge, the ability to flatten hand measurement, thumb palmar and radial abduction, and thumb MCP and interphalangeal joint range of motion. Furthermore, the outcome of revision surgery was assessed with the Conolly–Rath score (Conolly and Rath 1993) and finally the patients were asked to assess subjectively whether or not they had benefited from the revision surgery. Posteroanterior, oblique, and lateral radiographs were taken of the operated hands. From these radiographs, the scaphometacarpal space was measured and the radiographs were evaluated for MCP-joint hyperextension and residual bone fragments in the operative area.

### Statistics

Risk factors for revision surgery were analyzed with a conditional mixed model. There were 212/930 patients who had had surgery on both hands. Therefore, we used patients as random effects when estimating risk factors for revision surgery. Age was categorized according to quartiles since the impact of age on revision was nonlinear. The type of surgery was categorized into LRTI + bone channel, LRTI without bone channel, and other (all other primary surgery techniques) and immobilization time postoperatively was dichotomized at 0–4 weeks or 5–8 weeks. Variables were entered into the multivariable conditional mixed model one by one if their p-value in the univariable model was < 0.3. A variable was left in the final model if its p < 0.05 or the change in the Pseudo-Likelihood function was significant compared with the previous model. The results of the conditional mixed model are presented as odds ratio (OR) with a 95% confidence interval (CI). 2-tailed p-values are presented. The odds ratios can be interpreted as relative risks due to the small incidence of revision surgery (5%).

For simple correlations, Spearman’s correlation coefficient (rho) was calculated. Continuous data were analyzed with Student’s t-test or Welch’s t-test, the latter if Levene’s test showed unequal variances. Paired categorical data were analyzed with McNemar’s test.

SPSS for windows (IBM Corp. Released 2018. IBM SPSS Statistics for Windows, Version 25.0; IBM Corp, Armonk, NY, USA) and SAS (version 9.4; SAS Institute Inc, Cary, NC, USA) were used for analyses.

### Ethics, funding, and potential conflicts of interest

The institutional review board and ethical committee of Helsinki University Hospital, Finland approved the study, Dnro 6/13/03/02/2013. This research was funded by the University of Helsinki and the Department of Hand Surgery, Helsinki University Hospital, the Finnish Medical foundation, and Vappu Uuspää foundation. The authors report no conflicts of interest.

## Results

Altogether 50 patients (52 thumbs) had revision surgery (revision rate 4.6%). The revision rate was 4.7% for the LRTI group and 3.8% for the LRTI + bone tunnel group. In group 3 there were 4 revisions in 45 cases (Table 2). The total number of revisions performed was 65 with 9 patients having had multiple revision procedures for the same thumb (range 2–5 procedures) (Table 3, see Supplementary data). The mean time from primary surgery to the first revision procedure was 23 months (8–92).

**Table 2.** Primary procedures and revisions

Primary procedures	Number of primary surgeries	Number of revisions (%)
Conventional arthroplasty		
LRTI with APL tendon	679	32 (4.7%)
LRTI with APL tendon + bone tunnel	418	16 (3.8%)
Others		4^a^
LRTI with FRC tendon	2	0
LRTI with EPB tendon	2	0
Interposition with palmaris longus tendon		
without ligament reconstruction	5	0
Simple trapeziectomy	29	2
Partial trapeziectomy	7	2
Total number of procedures	1,142	52
Total number of patients	930	50

a revision rate not calculated due to small total number of operated patients.

The indication for revision surgery was pain in all cases, which was typically related to hand use and could be provoked at the outpatient clinic by loading the thumb axially towards the scaphoid, medially towards the trapezoid, or laterally stretching the joint capsule.

**Figure 1.**
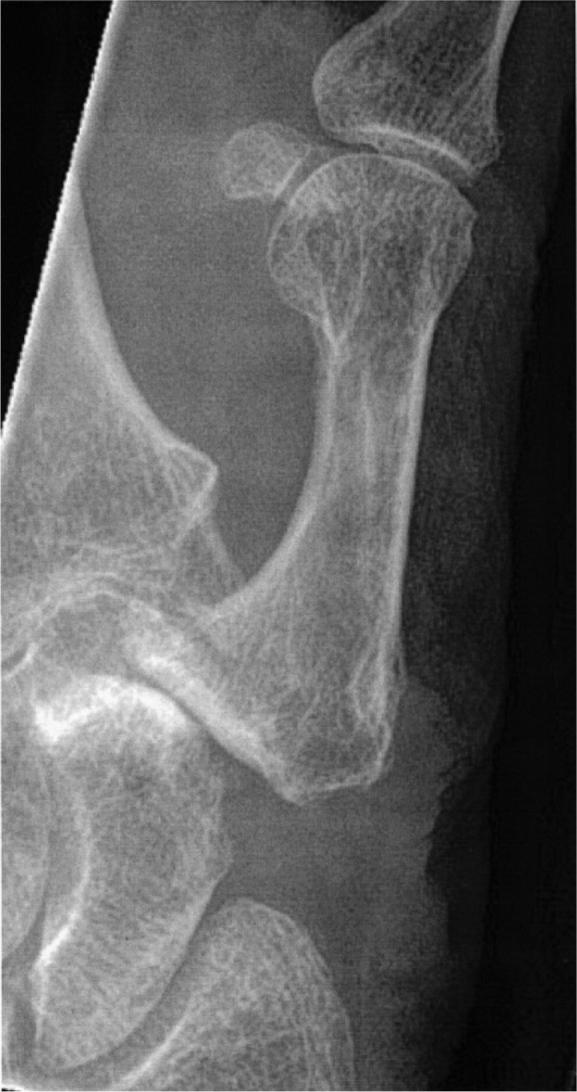
Subsidence of the metacarpal against the scaphoid 3 years after LRTI with APL.

**Figure 2.**
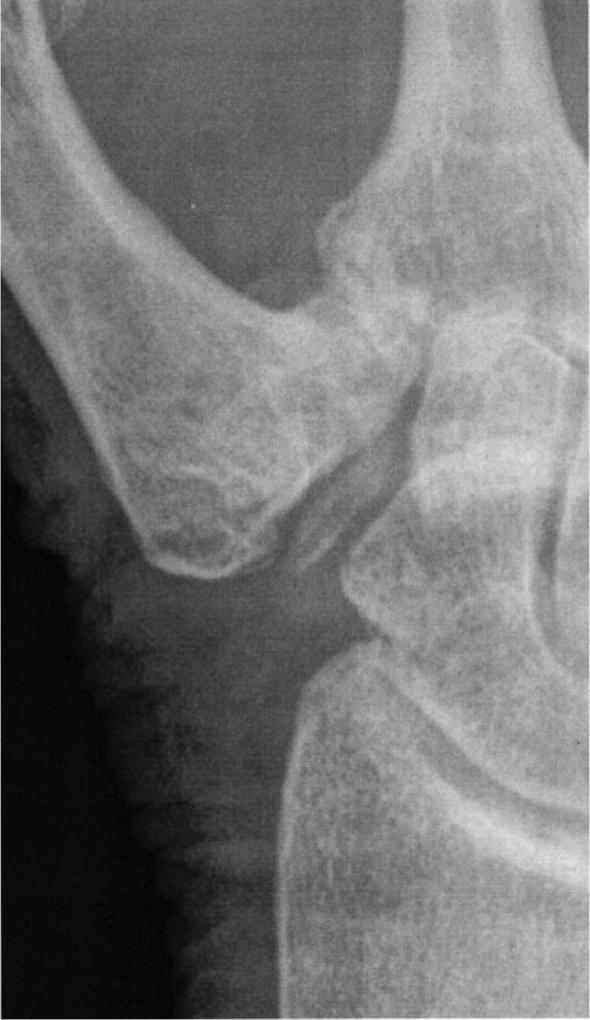
A remnant of the trapezium in the resection cavity after LRTI with APL.

In 30 cases, the main reason for pain as judged by the treating physician was subsidence of the metacarpal against the scaphoid or trapezoid bones (Figure 1) or contact of the metacarpal with remnants of the trapezium (Figure 2) in the resection cavity. In 13 cases, instability of the base of the metacarpal associated with MCP-joint hyperextension was the reason for the pain. In 2 cases (both hands of the same patient) the pain was caused by carpal instability. Pain related to tendon irritation or tenosynovitis was the reason for pain in 3 cases with tenderness of the APL tendon in the operative area or the distal FCR tendon related to irritation by the APL sling. Pain related to radial sensory nerve irritation or neuroma in 4 cases was provoked by touch or pressure with a positive Tinel sign.

Several techniques were used in revision cases (Table 4, see Supplementary data). For cases of metacarpal subsidence (Figure 2) the most common technique was interposition of a strip of fascia lata, suspension, and tendon interposition. In cases of instability of the thumb base and MCP-joint hyperextension, suspension arthroplasty and MCP-joint fusion were the most common procedures. Tenosynovitis of the FCR tendon caused by the APL sling was treated with FCR tenotomy or release of the sling. Neuromas were treated by release from scar tissue. No neuroma resections or nerve reconstructions were performed.

Patient age ≤ 55 years was a risk factor for revision surgery (9% revision rate) compared with age groups 56–60 years (OR 0.4, p = 0.02) (4% revision rate), 61–65 years (OR 0.1, p < 0.001) (1.2% revision rate) and > 66 years (OR 0.40, p = 0.02)(4.6% revision rate). There was a negative risk for revision surgery in patients operated on both thumbs at some point during the follow-up period (OR 0.4, p = 0.02). Furthermore, revision surgery was a rare event on the second operated hand (2 cases out of 202) if the operation on the first operated hand was successful. There was no statistically significant difference between the revision risk of LRTI with APL compared with LRTI with APL + bone tunnel (OR 0.8, p = 0.5) (Table 5). Comparison of age, sex, failed primary revision procedure, or the radiographic data obtained before revision surgery (scaphometacarpal space and MCP-joint hyperextension) with the outcome variables QuickDash, PEM, pain VAS, and key pinch showed that key pinch strength was statistically significantly higher in cases with a scaphometacarpal space ≤ 1mm (Table 6, see Supplementary data). A comparison of the radiographic data (scaphometacarpal space and MCP-joint hyperextension) between pre-revision and final follow-up showed fewer cases with a completely lost scaphometacarpal space (0–1mm) in the final follow-up radiographs (6/36) compared with pre-revision radiographs (11/42), but the difference was not statistically significant (p = 0.5). The number of cases with MCP-joint hyperextension in radiographs did not change significantly (p = 0.3). The mean pain VAS score was 40 mm (0–100), the mean DASH score 37 (2–73), and mean grip strength 23 kg (5–48). According to the Connolly–Rath score, there were 4 good, 27 fair, and 5 poor results. Regarding patient satisfaction, 31 of 34 patients felt subjectively that they had benefited from revision surgery. See Table 7 (Supplementary data) for all the results of revision surgery.

**Table 5.** Risk factors for revision surgery

	Univariable conditional mixed model		Multivariable conditional mixed model	
Risk factor	Odds ratio (95% CI)	p-value	Odds ratio (95% CI)	p-value
Surgery on both thumbs (negative risk)	49 (0.25–0.96)	0.04	0.42 (0.21–0.87)	0.02
Age, years				
≤ 55	1.0		1.0	
56–60	0.43 (0.21–0.88)	0.02	0.43 (0.21–0.87)	0.02
61–65	0.12 (0.04–0.42)	< 0.001	0.12 (0.04–0.41)	< 0.001
> 66	0.40 (0.19–0.87)	0.02	0.34 (0.15–0.76)	0.01
Sex	1.4 (0.55–3.8)	0.5	^a^	
Operated side	0.87 (0.50–1.5)	0.6	^a^	
Surgeon experience	1.04 (0.58–1.9)	0.9	^a^	
Surgical method				
LRTI APL	1.0		^a^	
LRTI APL + bone tunnel	0.81 (0.43–1.5)	0.5		
Others	2.0 (0.64–6.1)	0.2		
Complications of primary surgery	0.83 (0.25–2.8)	0.8	^a^	
Immobilization time after surgery	1.6 (0.82–3.3)	0.2	^a^	
Simultaneous surgery on the metacarpophalangeal joint	0.70 (0.09–5.4)	0.7	^a^	
Other simultaneous surgeries	0.60 (0.21–1.7)	0.3	^a^	

a Odds ratio not calculated because no significance in univariable model.

## Discussion

In our study, the revision rate for TMC arthroplasty was 4.6%, close to that of previous studies (2.6–4.0%) (Cooney et al. 2006, Megerle et al. 2011, Wilkens et al. 2017). Although generally only 1 revision is required (Cooney et al. 2006, Megerle et al. 2011), repeat revision procedures are not uncommon and an average of 5 procedures were required in 1 study (Renfree and Dell 2002). In our study, 9 of 50 patients had more than 1 revision procedure. Pain caused by metacarpal subsidence or instability seems to be the reason for revision surgery in almost all cases in the literature (Cooney et al. 2006, Megerle et al. 2011, Papatheodorou et al. 2017, Wilkens et al. 2017), which is similar to our findings.

Both good (Cooney et al. 2006) and poor (Megerle et al. 2011) results have been reported after revision TMC arthroplasty. The majority of our patients had a Conolly score of fair and said they benefited from the revision surgery. However, there were 5 poor results in 38 patients, which is approximately in line with previous studies (Cooney et al. 2006, Megerle et al. 2011). Regarding the subjective outcomes the mean QuickDASH score in our study of 38 and the mean Pain VAS score of 42 are in the same range as previously reported for revised patients after TMC arthroplasty (Megerle et al. 2011, Sadhu et al. 2016). Our results and the literature show that the majority of patients seem to benefit from revision surgery but the final outcome may still be worse than that of non-revised patients (Sadhu et al. 2016). The results of cases revised multiple times were not worse than those revised only once. Therefore, it seems that it is beneficial to operate on these patients several times if necessary to achieve a decent outcome.

Age ≤ 55 years was found to be a risk factor for revision with 9% of patients in this age group having revision surgery. In patients who have had a successful arthroplasty on the first thumb, the incidence of revision surgery on the contralateral second operated thumb was low (1%). Risk factors for revision surgery have been analyzed in only 2 studies (Cooney et al. 2006, Wilkens et al. 2017). Patient age, type of primary surgery, and surgeon experience were identified as risk factors for revision by Wilkens et al. but Cooney et al. did not identify any significant risk factors. Our study showed a similar result regarding age. This is probably related to the higher physical demands of younger patients, leading to more mechanical problems. The type of primary surgery in our study was not a risk factor for revision probably because the trapezium was completely removed in both of the main primary procedures groups (LRTI and LRTI + bone tunnel). In the study of Wilkens et al. the primary procedures included partial trapeziectomies and implant procedures, which are generally at higher risk for revision (Muermans and Coenen 1998, Richard et al. 2014, Wajon et al. 2015). In patients having an operation on both hands, the reason for the low incidence of revision procedures on the second operated hand could be related to ligament laxity affecting joint stability and possibly increasing the risk for metacarpal subsidence, which is involved in many revision cases (Megerle et al. 2011, Papatheodorou et al. 2017, Wilkens et al. 2017). Also factors such as patient confidence in the procedure being a good choice and that there is a good indication for surgery might explain this difference.

A large variety of procedures have been used for revision of TMC arthroplasty (Cooney et al. 2006, Megerle et al. 2011, Papatheodorou et al. 2017, Wilkens et al. 2017). No previous studies have been able to identify any correlation between outcome data and the type of revision procedure performed. This is probably related to the large number of different procedures in use for revision surgery, which leads to small subgroups of procedures that limit the statistical power of the analysis. This same problem applied to our study, making statistical analysis of the correlation between the type of revision procedure and final outcome unreliable. Analysis of radiographs taken at final follow-up showed that the scaphometacarpal space was lost completely in 6/36 cases. This may be because none of the surgical methods used for revision in our study are able to adequately address the problem of scaphometacarpal impingement.

One of the strengths of this study was the analysis of a large number of primary TMC arthroplasties performed for OA. This provided considerable statistical power to the analysis of risk factors for revision. However, because revision surgery is a rare event, there still was a limited number of revised patients, which made the statistical analysis of several potential factors affecting the final outcome unreliable. Also, a limitation of this study is the retrospective design, which introduces a risk of bias. In our hand surgical unit, trapeziectomy with and rarely without ligament reconstruction and interposition was the method of choice during the study period. However, in some selected cases for high-demand patients, who could be at high risk for revision, alternative methods such as implant arthroplasties were performed, which were excluded from the analysis due to the small number of cases.

In summary, patients ≤ 55 years are at greater risk for revision than older age groups. Revision surgery on the second operated hand after successful surgery on the first hand is rare. Repeat revision procedures are sometimes required but the outcome does not differ from patients undergoing only one revision. A bone tunnel (LRTI + bone tunnel) to stabilize the thumb base does not reduce the risk of revision surgery compared with other surgical methods used in this study.

### Supplementary data

Tables 3, 4, 6, and 7 are available as supplementary data in the online version of this article, http://dx.doi.org/10.1080/17453674.2019.1599253

SM composed the manuscript, gathered the data, and met the patients at the follow-up visit. EW contributed substantially to the design of the study, the composition of the manuscript, and planning of the data acquisition.

The authors wish to thank statistician Pasi Ohtonen, MSc, for consultation with the statistical analysis in this study.

*Acta* thanks Philippe Kopylov for help with peer review of this study.

## Supplementary Material

Supplemental Material

## Supplementary data

Tables 3, 4, 6, and 7 are available as supplementary data in the online version of this article, http://dx.doi.org/10.1080/17453674.2019.1599253
