# Supplementary material for: Revision of trapeziometacarpal arthroplasty: risk factors, procedures and outcomes
Source: Acta Orthop. 2019 Apr 1;90(4):389–93. doi: 10.1080/17453674.2019.1599253 (PMC6718170; doi:10.1080/17453674.2019.1599253)
Supplement: Supplemental Material [file IORT_A_1599253_SM6460.pdf]

Supplementary data

Table 3. Patients with multiple revision procedures

#	Primary procedure	Revision 1	Revision 2	Revision 3	Revision 4	Revision 5
1	LRTI with APL + bone tunnel	Adductor release	MCP fusion			
2	LRTI with APL	Tenotomy of APL sling	Soft tissue interposition (fascia lata)	Soft tissue interposition (fascia lata) revision	MC1–Scaphoid fusion	MC1–Scaphoid fusion (revision)
3	LRTI with APL + bone tunnel	Suspension MC1–MC2	Soft tissue interposition (fascia lata)			
4	LRTI with APL	Soft tissue interposition (fascia lata)	Removal of residual bone fragments			
5	Simple trapeziectomy	LRTI with APL + bone tunnel	Suspension + MCP fusion			
6	LRTI with APL + bone tunnel	Radiolunate fusion (both sides)	Total wrist fusion (left side)	MC1–MC2 fusion (left side)		
7	LRTI with APL	Removal of residual bone fragments	FCR tenotomy			
8	LRTI with APL + bone tunnel	Soft tissue interposition (fascia lata)	MCP fusion			
9	LRTI with APL + bone tunnel	Implant (RegJoint)	Soft tissue interposition (fascia lata)			

Table 4. Revision procedures

All revision procedures (primary and repeat procedures)	n
Soft tissue interposition with fascia lata	19
Soft tissue interposition with palmaris longus	4
Soft tissue interposition with joint capsule	1
LRTI with APL	1
LRTI with APL + bone tunnel	2
Suspension arthroplasty between MC1 and MC2 using autologous tendon	7
Suspension arthroplasty between MC1 and MC2 using TightRope	1
Debridement of resection cavity of residual bone fragments	8
Tenolysis (FCR, APL, adductor release)	6
Neurolysis of superficial branch of radial nerve	4
Thumb MCP joint fusion	3
Partial or total wrist fusion	4
MC1–Scaphoid fusion	2
Implant arthroplasty (RegJoint)	3
Total	65

 Acta Orthopaedica 2019; 90 (DOI: 10.1080/17453674.2019.1599253)

Table 6. Factors affecting final outcome of revision surgery

**Spearmans rho**<br>Age (not categorized)		p-value		Correlation coefficient	
DASH		0.5		rho –0.12	
PEM		0.5		rho –0.12	
pain VAS		0.6		rho –0.10	
Key pinch		0.5		rho –0.11	
**T-test**, mean, (SD) and [range]					Difference between means (95% CI)
Sex	Male		Female		
DASH	40 (18)	[21–55]	37 (20)	[2–73]	3 (–22 to 27)
PEM	36 (25)	[13–63]	44(24)	[3–94]	8 (–38 to 21)
pain VAS	47 (38)	[20–90]	42 (29)	[0–100]	5 (–31 to 41)
Key pinch	8 (1)	[7–9]	5 (3)	[2–11]	3 (–1 to 7)
Failed primary revision					
	Yes		No		
DASH	39 (13)	[20–61]	37 (21)	[2–73]	2 (–20 to 16)
PEM	40 (21)	[16–66]	44 (25)	[3–94]	4 (–18 to 26)
pain VAS	40 (28)	[0–70]	43 (30)	[0–100]	3 (–24 to 29)
Key pinch	5 (2)	[2–8]	5 (3)	[2–11]	0 (–2 to 3)
Scaphometacarpal space in radiograph					
	0–1 mm		> 1 mm		
DASH	45 (18)	[5–66]	37 (20)	[2–73]	8 (–8 to 24)
PEM	47 (16)	[25–66]	43 (27)	[3–94]	4 (–16 to 24)
pain VAS	47 (26)	[20–90]	37 (34)	[0–100]	10 (–16 to 35)
Key pinch	7 (3)	[3–10]	5 (3)	[2–11]	2 (0 to 5) [a]
MCP-joint hyperextension in radiograph					
	Yes		No		
DASH	44 (25)	[11–73]	38 (17)	[2–71]	6 (–21 to 10)
PEM	47 (30)	[3–94]	43 (21)	[3–87]	4 (–23 to 16)
pain VAS	39 (32)	[0–90]	41 (32)	[0–100]	2 (–25 to 28)
Key pinch	5 (2)	[2–8]	6 (3)	[2–11]	1 (–1 to 3)

[a] p-value = 0.04

Table 7. Results of revision surgery. Number of patients with data = 38

DASH [a]	37 (23–45)	[2 to 73]
PEM [a]	40 (27–63)	[3 to 94]
Pain VAS (mm) [a]	40 (20–70)	[0 to 100]
Key pinch (kg) [a]	5 (3–8)	[2 to 11]
Tip pinch (kg) [a]	3 (2–6)	[0 to 12]
Grip strength (kg) [b]	23 (9)	[5 to 48]
Kapandji score [b]	9 (2)	[3 to 10]
Ability to flatten hand (cm)	2 (1–4)	[0 to 6]
Palmar abduction (°) [b]	67 (15)	[30 to 95]
Radial abduction (°) [b]	81 (18)	[20 to 110]
MCP extension (°) [a]	30 (15–40)	[–30 to 60]
MCP flexion (°) [b]	39 (19)	[0 to 90]
IP extension (°) [a]	15 (0–20)	[–20 to 45]
IP flexion (°) [b]	69 (16)	[40 to 95]
Connolly Rath score (good/fair/poor)	4/27/5	
Benefit of surgery (yes/no)	31/3	
Scaphometacarpal space (mm) [a]	3 (2–4)	[0 to 7]
Adduction/MCP-joint hyperextension (yes/no)	12/27	
Bone fragments in operative area (yes/no)	5/31	

[a] Median (interquartile range) [range]
[b] Median (standard deviation) [range]
